# The neuro–immune–vascular landscape of lung cancer brain metastasis: insights from organoid models

**DOI:** 10.3389/fimmu.2026.1886585

**Published:** 2026-08-18

**Authors:** Jiaojiao Li, Mingjun Xu, Lei Wang, Yan Yu

**Affiliations:** Department of Oncology, Harbin Medical University Cancer Hospital, Harbin, China

**Keywords:** blood–brain barrier, brain metastasis, lung cancer brain metastasis, neuro–immune–vascular, organoids

## Abstract

Lung cancer brain metastasis (LCBM) is a major cause of cancer-related mortality, driven by the restrictive blood–brain barrier (BBB), an immunosuppressive microenvironment, and dynamic metabolic reprogramming. Increasing evidence suggests that LCBM is governed by coordinated interactions among neural, immune, and vascular components that regulate metastatic colonization, progression, and immune evasion. However, conventional models fail to recapitulate this multicellular, spatially organized ecosystem, limiting mechanistic understanding. Recent advances in organoid technology provide a new paradigm for modeling LCBM. Integrative platforms, including cerebral organoid assembloids, vascularized microfluidic systems, and BBB models, enable reconstruction of key components and dynamic interactions within the brain metastatic niche. These systems have uncovered critical regulatory axes, such as astrocyte-driven colonization, neurovascular remodeling, and metabolic–immune coupling. Beyond structural modeling, organoid platforms enable functional interrogation of patient-specific therapeutic responses in physiologically relevant contexts, facilitating drug sensitivity testing, immunotherapy optimization, and multi-modal treatment modeling. Despite current limitations, organoid-based models provide a critical framework for dissecting the neuro–immune–vascular landscape of LCBM and bridging mechanistic insights with clinical translation, offering new opportunities for therapeutic innovation and personalized treatment.

## 1 Introduction

Lung cancer remains the leading cause of cancer-related death, and is a major contributor to the development of brain metastases (BMs), accounting for 40%–50% of cases (1). Pathologically, lung cancer is primarily classified into two major subtypes: non-small cell lung cancer (NSCLC), which accounts for approximately 85%–88% of cases, and small cell lung cancer (SCLC), comprising 12%–15% (2). Approximately 20% of patients with NSCLC will present with BMs at the time of diagnosis and 25%–50% will develop BMs during disease progression (3). In SCLC, around 10% of patients have BMs at the time of diagnosis, while up to 50% will develop BMs over the course of the disease (4).

Historically, the brain has remained a challenging therapeutic target in metastatic lung cancer due to the presence of the blood–brain barrier (BBB) and the limited penetration of drugs into the central nervous system (CNS). Consequently, treatment of BMs has traditionally relied on local approaches, including surgical resection, stereotactic radiosurgery (SRS), and whole-brain radiation therapy (WBRT). Although these local treatment modalities can achieve a certain degree of intracranial disease control, their impact on systemic disease control and long-term survival remains limited.

In recent years, however, significant advances have been achieved in systemic therapies, particularly targeted therapies and immunotherapies (5). Immunotherapies alone or in combination with other therapies have shown promising efficacy in patients with BMs. Nevertheless, patients with LCBM exhibit a paradoxical pattern in PD-L1–positive populations (PD-L1 > 1%), characterized by favorable predictive responses but poor overall prognosis (6). This observation suggests that the brain metastasis–specific microenvironment may attenuate the long-term effectiveness of immunotherapy, highlighting the importance of further elucidating the underlying mechanisms of the brain metastasis.

Meanwhile, with the identification of oncogenic driver mutations and the development of next-generation tyrosine kinase inhibitors (TKIs), targeted therapies have also yielded encouraging intracranial activity in patients with actionable genetic alterations (AGAs) in clinical trials (7–10).

The complexity of lung cancer brain metastasis (LCBM) is reflected not only in heterogeneous treatment responses but also in its pronounced genetic heterogeneity across patients, characterized by diverse mutations and variability in key driver genes (11). LCBM is driven by distinct molecular alterations. In NSCLC, these commonly include mutations in epidermal growth factor receptor (*EGFR*), anaplastic lymphoma kinase (*ALK*), c-ros oncogene 1 (*ROS1*), and Kirsten rat sarcoma viral oncogene homolog (*KRAS*), whereas in SCLC, *MYC* amplification and tumor protein p53 (*TP53*) mutations are predominant (12). These molecular alterations not only influence the risk of brain metastasis but may also, to some extent, determine the response patterns of patients with BMs to systemic therapies.

Moreover, the complexity of LCBM lies not only in the marked heterogeneity of therapeutic responses but also in the distinct biological differences between the brain microenvironment and the primary tumor site. The brain tumor microenvironment (TME) imposes unique selective pressures on metastatic cells. To successfully colonize the brain, cancer cells must adapt to and dynamically interact with CNS–resident cells, as well as infiltrating immune populations. Within this context, immune modulation and vascular regulation play central roles in shaping metastatic progression (13, 14).

Patient-derived xenografts (PDXs), a widely used model for tumor research, preserve the histopathological architecture and genomic features of the original tumors. However, their application is limited by several drawbacks, including high cost, low engraftment efficiency, and prolonged development time (15, 16). These limitations pose significant challenges for translational research and therapeutic development.

Accordingly, there is a critical need for more physiologically relevant and scalable model systems that better capture tumor heterogeneity and microenvironmental complexity. In this context, organoid-based models of LCBM emerge as a promising approach. By recapitulating key features of tumor architecture and the TME, LCBM organoids enable the investigation of tumor–immune interactions and provide new opportunities for studying disease mechanisms and advancing personalized therapeutic strategies.

Given the rapid evolution and expanding applications of LCBM organoids, this review provides a comprehensive overview of their construction and recent advances over the past five years. We further discuss their emerging roles in modeling neuro–immune–vascular interactions and their potential in precision immuno-oncology, particularly in the context of immunotherapy, and highlight future directions for improving the understanding and treatment of LCBM.

## 2 Blood–brain barrier and brain tumor microenvironment

### 2.1 Blood–brain barrier remodeling and metabolic adaptations

LCBM is increasingly recognized as a dynamic neuro–immune–vascular ecosystem in which BBB remodeling, metabolic adaptation, and immune reprogramming are tightly interconnected rather than independent processes. The BBB is composed of a tightly connected monolayer of brain microvascular endothelial cells (BMECs), supported by astrocytes and pericytes. These components form a highly regulated neurovascular unit that maintains homeostasis, influences normal brain function, and protects the brain from potential toxins and pathogens. In brain tumors and metastases, BBB integrity is frequently disrupted, leading to the formation of the blood–brain tumor barrier (BBTB) (17). Rather than being a passive process, successful BM requires tumor cells to actively adapt to the unique neuro–immune–vascular landscape of the CNS (18). Lung tumor cells and TME cells secrete angiogenic and extracellular matrix (ECM) degradation factors that function to increase BBB permeability and facilitate tumor cell extravasation into the brain (19).

Once lung tumor cells extravasate and settle in the brain, they must overcome the impact of the brain microenvironment. To survive and expand within this niche, metastatic cells undergo adaptive metabolic reprogramming (20, 21). Multi-omic profiling studies have demonstrated that oxidative phosphorylation (OXPHOS) is significantly enhanced in LCBM and other BM types compared with patient-matched primary tumors or extracranial metastases, reflecting a shift in energy metabolism associated with the unique constraints of the brain microenvironment (22). In parallel, 3-phosphoglycerate dehydrogenase (PHGDH) emerges as a key determinant of brain metastasis in NSCLC and other cancer types. Consistently, PHGDH-dependent serine biosynthesis supports metastatic fitness under serine-limited conditions (23). More recently, brain-specific metabolic adaptation has been linked to neurotransmitter metabolism. In NSCLC, dysregulation of the forkhead box A2 (FOXA2)/aminotransferase (ABAT) axis leads to γ-aminobutyric acid (GABA) accumulation, which promotes astrocyte-mediated microenvironmental remodeling, thereby facilitating BM colonization (24). Emerging evidence further suggests that metabolic reprogramming in BMs is tightly coupled with immune-mediated niche remodeling. Neutrophil extracellular traps (NETs) drive fatty acid metabolic reprogramming within the metastatic niche, leading to increased palmitic acid accumulation and enhanced metastatic colonization in NSCLC (25).

Key metabolic pathways, including glucose, fatty acid and amino acid metabolism, are co-opted not only to sustain tumor cell survival and growth but also to regulate interactions with resident brain cells, thereby reshaping the metastatic microenvironment. Notably, proliferative and dormant metastatic cells within the brain exhibit distinct metabolic profiles, highlighting the complexity of therapeutic targeting (26). Collectively, these findings suggest that BBB remodeling and metabolic adaptation are tightly interconnected processes that enable tumor cell survival in the brain and further shape the local immunosuppressive microenvironment.

### 2.2 Neuro–immune–vascular interactions in brain tumor microenvironment

The brain TME comprises neurons, immune cells, and vascular elements, collectively forming a specialized niche that regulates tumor seeding and progression (27). Across studies of human BM, T cells and macrophages represent the dominant immune cell populations, regardless of the primary tumor type (28). However, compared to matched primary lung tumors, LCBM lesions are characterized by a “cold” immunophenotype, exhibiting diminished PD-L1 expression and sparse CD8^+^ T-cell infiltration, which underscores a potent immunosuppressive milieu (29).

#### 2.2.1 Local immune suppression driven by myeloid and lymphoid reprogramming

In this context, multiple immune cell populations cooperate to shape immune evasion. Infiltrating monocytes differentiate into macrophages, which in NSCLC-derived BMs further polarize into metastasis-associated macrophages, constituting the majority of macrophages within metastatic foci (30). Microglia, the brain resident macrophages, are the major immune cell type that modulates disseminated cancer cells (DCCs) to adapt to the brain TME. Bone-marrow-derived myeloid cells (BMDMs) cooperate with microglia to regulate brain responses in LCBM. Notably, Guldner et al. demonstrated that CNS-resident microglia, rather than BMDMs, promote brain metastatic outgrowth (31).

In parallel, dendritic cells (DCs), which are essential for antigen presentation and T-cell priming, frequently exhibit functional impairment in the BM niche, leading to defective activation of cytotoxic T cells (32). Concurrently, immunosuppressive lymphoid populations further reinforce immune escape. Regulatory T cells (Tregs) are often enriched within the brain metastatic microenvironment, where they suppress effector T-cell function and promote immune tolerance (33). In addition, dysregulation of immune checkpoint pathways, including V-domain Ig suppressor of T cell activation (VISTA) and PD-L1 signaling, further reinforces local immune suppression and facilitates metastatic progression. Neutrophils also contribute to BM immune remodeling through the formation of neutrophil extracellular traps (NETs), which impair antitumor immunity and promote tumor progression (25).

#### 2.2.2 Astrocyte-centered neuro–immune regulation

Beyond immune cell reprogramming, neural-associated stromal cells play a critical role in shaping the metastatic niche. Among the various cellular components, astrocytes emerge as central regulators of the brain metastatic niche, orchestrating both immune suppression and tumor adaptation. Microglia accumulate around metastatic foci and exert both pro- and anti-metastatic effects, whereas astrocytes dominate the stromal compartment of BMs (34). As the predominant stromal cell type in the brain, astrocytes play a central role in metastatic niche formation. Bidirectional interactions between astrocytes and tumor cells contribute to immune suppression and tumor survival.

#### 2.2.3 Spatial and vascular remodeling of the brain metastatic niche

Recent multi-omic studies highlight the spatial heterogeneity and vascular remodeling of the brain metastatic microenvironment. Spatial transcriptomic analyses show that the brain TME undergoes extensive remodeling, leading to the formation of an immunosuppressive and fibrotic niche characterized by impaired antigen presentation, dysfunction of B and T cells, increased neutrophil infiltration, enrichment of M2-like macrophages, and activation of reactive astrocytes (35). Furthermore, single-cell and bulk RNA sequencing of vascular cells demonstrate that endothelial and mural cells acquire immunoregulatory phenotypes that contribute to immune suppression (36). In addition, early metastatic epithelial cell clusters enriched in BMs display distinct spatial programs and active immune crosstalk, suggesting that metastatic priming and niche establishment may occur within specialized microenvironmental regions rather than through uniform tumor expansion (37).

Functional vascular remodeling in BMs is governed not only by structural reorganization but also by dynamic biochemical and biomechanical signaling (38). Alterations in BBB integrity and vascular permeability create a permissive microenvironment that amplifies endothelial activation (39). Activated brain endothelial cells can then reshape the metastatic niche through a coordinated angiocrine and inflammatory signaling program. This includes the release or regulation of vascular-associated cytokines and growth factors, such as vascular endothelial growth factor (VEGF), fibroblast growth factor (FGF), and transforming growth factor-β (TGF-β), as well as the upregulation of adhesion molecules and chemokines, including ICAM-1, VCAM-1, selectins, CXCL10, CXCL12, and CCL2. Together, these signals promote angiogenic remodeling and regulate the adhesion, crawling, and transendothelial migration of circulating T cells, monocytes, neutrophils, and dendritic cells (40, 41).

However, this vascular activation does not necessarily translate into effective antitumor immunity. In the brain metastatic niche, endothelial and mural cells can acquire immunosuppressive phenotypes, including increased expression of vascular immune checkpoints such as CD276/B7-H3, thereby restricting cytotoxic T-cell infiltration and reinforcing immune exclusion (36). Pericytes further modulate this process by controlling endothelial transcytosis, tight-junction stability, capillary diameter, and local blood flow. Pericyte detachment or phenotypic reprogramming may therefore increase vascular leakage while simultaneously generating a perivascular niche that protects metastatic cells from immune surveillance (42). In addition, altered hemodynamic forces within abnormal tumor vessels, including changes in shear stress and heterogeneous perfusion, influence endothelial phenotype, tight-junction organization, angiogenic sprouting, leukocyte margination, and immune-cell extravasation (43). Thus, vascular remodeling links BBB dysfunction, angiocrine signaling, neuronal stress responses, immune-cell trafficking, and glial activation, providing a direct mechanistic bridge between the vascular, immune, and neural components of the LCBM microenvironment.

#### 2.2.4 Brain-wide immune regulation by the meningeal lymphatic system

Beyond local cellular and vascular interactions, brain-wide immune regulation also contributes to metastatic progression. Recent evidence uncovers the critical role of the meningeal lymphatic system in orchestrating immune surveillance during BMs. Zhang et al. utilized optimized MRI protocols to demonstrate significant structural and functional disintegration of meningeal lymphatic vessels adjacent to the superior sagittal sinus in both murine models and patients with LCBM. This disruption leads to impaired lymphatic drainage to the deep cervical lymph nodes, particularly those at level IIA, thereby compromising antigen presentation and immune cell trafficking in the brain. Crucially, the extent of mLV impairment directly correlates with disease progression and diminished efficacy of intrathecal chemotherapy (44). These findings delineate a previously underappreciated lymphatic-immune axis, suggesting that the collapse of the meningeal lymphatic system is a key driver in establishing the immunosuppressive sanctuary of LCBM.

**Figure 1:**
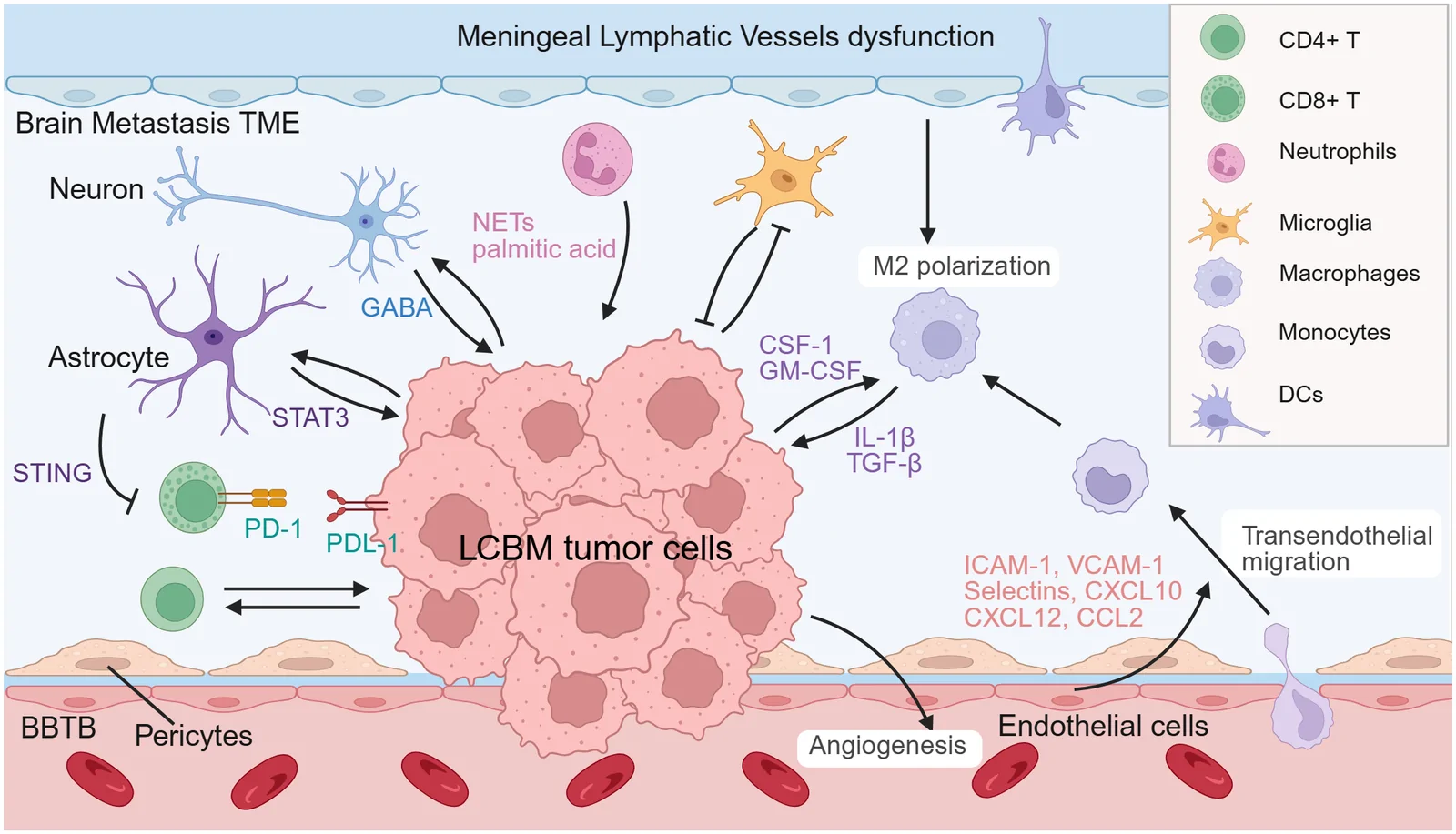
Neuro–immune–vascular interactions in the brain metastasis tumor microenvironment. This schematic summarizes the neuro–immune–vascular interactions that shape the brain metastasis tumor microenvironment (TME) in lung cancer brain metastasis (LCBM). Tumor cells interact with astrocytes, neurons, microglia, macrophages, monocytes, neutrophils, dendritic cells (DCs), T cells, endothelial cells, and pericytes. Astrocytes promote tumor progression through STAT3- and STING-associated signaling, while immune checkpoints such as programmed cell death protein 1/programmed death-ligand 1 (PD-1/PD-L1) suppress CD8^+^ T-cell activity. Myeloid cells and neutrophils contribute to immune suppression through macrophage polarization, cytokine release, neutrophil extracellular trap (NET) formation, and metabolic remodeling. Endothelial cells and pericytes regulate blood–brain tumor barrier (BBTB) remodeling, angiogenesis, and immune-cell transendothelial migration. Meningeal lymphatic vessel dysfunction further impairs immune surveillance. Together, these interactions promote immune evasion and LCBM progression.

Collectively, these findings indicate that the immunosuppressive landscape of BMs is orchestrated by coordinated interactions among immune, stromal, and vascular components. This integrated network limits antitumor immunity while promoting metastatic progression and therapeutic resistance, supporting a model of coordinated neuro–immune–vascular regulation rather than isolated pathways (**Figure 1**). However, most current evidence describing the neuro–immune–vascular landscape of LCBM is derived from end-point histological analyses, bulk or single-cell profiling, and murine models. Although these approaches have revealed important cellular and molecular alterations, they provide limited capacity to reconstruct dynamic interactions among metastatic tumor cells, brain-resident stromal cells, vascular components, and immune populations in a human-specific context. Moreover, many reported mechanisms remain associative rather than causally validated. These limitations underscore the need for human-relevant experimental platforms that can functionally model the temporal evolution of the LCBM niche.

## 3 Advanced LCBM organoid models

### 3.1 Evolution from single organoids to LCBM-relevant assembloids

**Figure 2:**
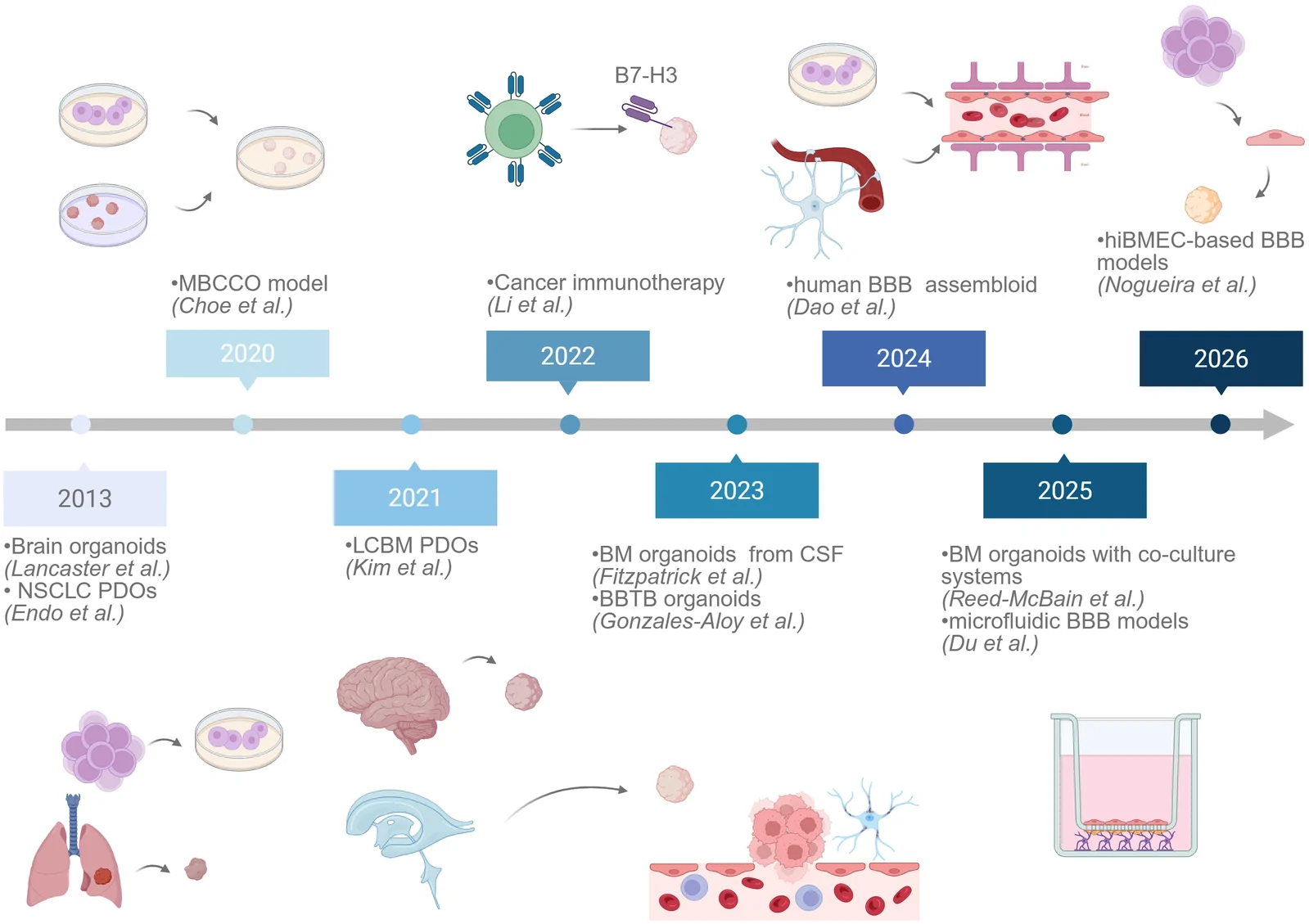
Evolution of organoid models for LCBM. This timeline illustrates the development of organoid models relevant to LCBM research, from early brain organoids and non-small cell lung cancer (NSCLC) patient-derived organoids (PDOs) to metastatic brain cancer cerebral organoids, LCBM PDOs, cerebrospinal fluid (CSF)-derived brain metastasis organoids, blood–brain barrier/blood–brain tumor barrier (BBB/BBTB) models, immunotherapy-related organoid systems, human BBB assembloids, and microfluidic BBB platforms. These advances reflect a transition from simple three-dimensional cultures to integrated multicellular systems that better model tumor–brain interactions, vascular barriers, immune responses, and therapeutic testing.

Organoid technologies have rapidly evolved over the past decade, transitioning from simple three-dimensional (3D) cultures to complex, multi-component systems capable of recapitulating the brain metastatic niche, as illustrated in **Figure 2** (45). In 2013, Lancaster et al. pioneered the establishment of brain organoids derived from embryonic stem cells (ESCs), successfully recapitulating key aspects of human cortical development (46). Concurrently, Endo et al. established patient-derived organoids (PDOs) from surgical specimens and pleural effusions of patients with NSCLC, achieving a success rate of approximately 80% (47).

### 3.2 Tumor–brain interaction modeling using co-culture systems

To address the lack of tumor–brain interaction in early organoids, co-culture systems were developed to integrate cancer cells with brain-like organoids. Choe et al. developed a 3D *in vitro* model, termed the metastatic brain cancer cerebral organoid (MBCCO), by integrating human cancer cells with embryonic stem cell–derived cerebral organoids. This system recapitulates key features of LCBM, including tumor cell adhesion, proliferation, migration, and cell–cell interactions, and supports drug screening applications (48). Kim et al. established PDOs with surgical samples from LCBM patients and successfully performed patient-specific drug testing and validation of preclinical models, thereby demonstrating the feasibility of preclinical investigations based on PDOs (49). Fitzpatrick et al. established five PDOs of breast cancer with leptomeningeal metastasis by optimizing the *in vitro* expansion method for extracting a small number of disseminated cancer cells from the patients’ cerebrospinal fluid (CSF) (50). This approach highlights the feasibility of generating LCBM organoids from cerebrospinal fluid samples, providing a promising strategy for modeling leptomeningeal metastasis. Importantly, these models allow for the investigation of bidirectional signaling between metastatic seeds and the brain parenchyma, a process largely inaccessible in isolated tumor cultures.

### 3.3 Emergence of neuro–immune–vascular organoid systems

Recent breakthroughs have transitioned organoid modeling toward integrated, multi-lineage systems that capture the spatial complexity and stromal components of the brain microenvironment. Building upon these advances, microfluidic platforms enable the development of spatially organized co-culture systems. By integrating tumor cells with induced pluripotent stem cell (iPSC)-derived cerebral organoids (composed of neurons and astrocytes) without physical separation between tumor and stromal compartments, these systems recapitulate tumor–neural interactions, providing a physiologically relevant framework for modeling metastatic colonization (51). Furthermore, the emergence of vascularized organoid-on-a-chip systems allows for the recruitment of peripheral immune populations. By reconstructing the neurovascular interface, these platforms can simulate the infiltration of T cells and neutrophils from the circulation into the brain parenchyma (52). This capability is crucial for studying how the LCBM niche orchestrates T-cell exhaustion and the formation of NETs, which have been identified as key drivers of metastatic fitness and immune evasion in the brain microenvironment (53). Complementing these engineered “recruitment” models, the Individualized Patient Tumor Organoid (IPTO) system represents an alternative paradigm by preserving the endogenous immune landscape already present within the metastatic tissue. As demonstrated by Peng et al., integrating patient-derived tumor explants into hiPSC-derived cerebral organoids not only maintains the native histopathological architecture and intratumoral heterogeneity but also retains resident immune populations that are often lost in conventional models (54). More recently, human organoid-based co-culture systems have emerged as powerful platforms for modeling metastatic colonization by reconstructing organ-specific microenvironments (55).

### 3.4 Engineering the blood–brain barrier and vascular niche

A major frontier in LCBM modeling is the functional reconstruction of the BBB and its pathological transformation into the BBTB. To address this challenge, microfluidic organ-on-a-chip systems enable the integration of endothelial cells, pericytes, and astrocytes to model dynamic vascular barriers and quantify drug permeability (17). Human BBB assembloids further recapitulate molecular and functional features of neurovascular units, including neurovascular crosstalk and spatial organization (56). In parallel, hiPSC-derived brain microvascular endothelial cell systems provide mechanistic insights into BBB development through Wnt/β-catenin signaling (57). Vascularized tumor organoid-on-a-chip platforms further reveal that metastatic tumor cells actively drive angiogenesis and vascular migration via Notch signaling, linking vascular remodeling with metastatic potential (58). Nevertheless, current LCBM organoid systems should not be viewed as complete replicas of the brain metastatic microenvironment. Most models still incompletely incorporate mature vascular barriers, functional immune trafficking, meningeal lymphatic drainage, and region-specific neural maturation. Therefore, their major value lies not in replacing animal models or clinical specimens, but in providing controllable, human-relevant platforms for testing specific mechanistic hypotheses. This distinction is important when interpreting organoid-derived findings, particularly for mechanisms involving immune surveillance, BBB permeability, and vascular remodeling. To provide a comprehensive overview, **Table 1** summarizes the key features, advantages, and limitations of different organoid culture systems used to model LCBM.

**Table 1:** Comparison of organoid models in LCBM research.

Model type	Cellular components	Key advantages	Limitations	References
PDOs	Patient-derived tumor cells	Preserve tumor heterogeneity and genomic features; suitable for personalized drug testing(chemotherapy and targeted therapy)	Lack tumor microenvironment (immune, vascular components); dependence on exogenous growth factors; lack of prospective validation	(49)
Cerebral organoids	Neurons, astrocytes, neural progenitors	Recapitulate brain developmental architecture and neural context; enable modeling of neurodevelopmental diseases	Immature cell populations; absence of tumor and vascular components	(46)
MBCCO model	Cell-line-derived tumor cells, neurons, astrocytes, neural progenitors	Recapitulate tumor–brain interactions and metastatic colonization; drug response studies	Restricted to early metastatic niche formation; poor suitability for high-throughput screening	(48)
IPTOs	Patient-derived tumor cells; neurons; astrocytes; neural progenitors	High patient tumor fidelity; ideal for precision medicine	Low reproducibility; high sample variability; prolonged culture window	(54)
Immune-integrated organoids	Tumor cells, immune cells (T cells, microglia, macrophages	Model tumor–immune interactions and immunosuppressive microenvironment	Lake of vascular and lymphatic systems; limitied immune trafficking; short lifespan of immune cells; reduced standardization and reproducibility	(91)
Vascularized organoids	Tumor cells, endothelial cells, pericytes	Mimic angiogenesis and partial BBB features; improving physiological relevance of tumor microenvironment	Incomplete vascular functionality; insufficient maturation of BBB-like structures	(52)
Organ-on-a-chip systems	Tumor cells, BB/TME cells, microfluidics	Dynamic flow control; precise spatial organization; BBB modeling	Limited scalability and throughput; lack of standardized platforms; high technical requirement	(58)

BBB, blood–brain barrier; LCBM, lung cancer brain metastasis; MBCCO, metastatic brain cancer cerebral organoid; PDOs, patient-derived organoids; IPTOs, individualized patient tumor organoid.

## 4 Organoid-based dissection of neuro–immune–vascular crosstalk in LCBM

**Figure 3:**
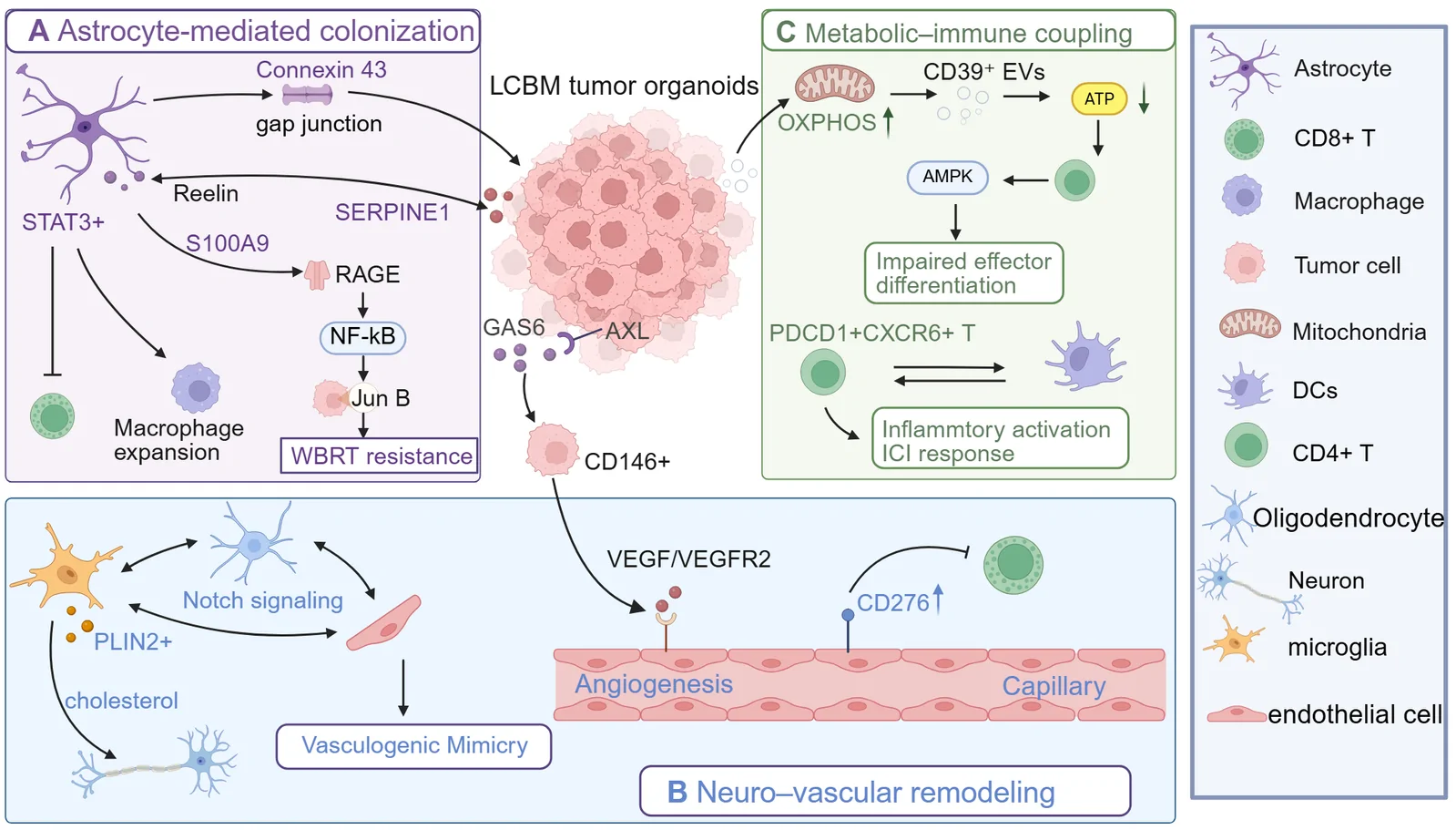
Organoid-based dissection of neuro–immune–vascular mechanisms in LCBM. This schematic summarizes key mechanisms of LCBM revealed by organoid-based models. Astrocyte–tumor crosstalk promotes metastatic colonization through connexin 43-mediated gap junctions, Reelin–SERPINE1 signaling, STAT3 activation, and the S100A9–receptor for advanced glycation end-products (RAGE)–NF-κB/JunB axis. Neurovascular remodeling involves GAS6–AXL–CD146 signaling, vascular endothelial growth factor/vascular endothelial growth factor receptor 2 (VEGF/VEGFR2)-mediated angiogenesis, CD276-associated immune exclusion, and Notch-related vasculogenic mimicry. Metabolic–immune coupling is characterized by increased oxidative phosphorylation (OXPHOS), CD39^+^ extracellular vesicle (EV) release, adenosine triphosphate (ATP) depletion, and AMP-activated protein kinase (AMPK) activation in CD4^+^ T cells, leading to impaired immune function and therapy resistance.

Recent organoid-based studies indicate that LCBM progression is governed by a hierarchically organized neuro–immune–vascular network, integrating astrocyte-driven tumor reprogramming, neurovascular immune regulation, and metabolic–immune coupling (**Figure 3**). Advances in organoid technology have enabled the incorporation of key non-neuronal components, including astrocytes, microglia, and vascular cells, thereby enhancing the physiological relevance of these models. Together, these systems provide a unified mechanistic framework in which interconnected regulatory axes drive tumor progression and immune evasion, as summarized in **Table 2**.

**Table 2:** Emerging mechanisms and therapeutic targets revealed by organoid models in LCBM.

Functional axis	Key mechanisms	Biological role	Therapeutic implications	References
Astrocyte–tumor interaction	Connexin 43 gap junctions; cGAMP–STING signaling	Promote tumor survival, proliferation, and immune modulation	Target gap junction signaling; STING pathway modulation	(48, 59)
Astrocyte-driven resistance	S100A9–RAGE–NF-κB axis	Induces radioresistance and stem-like phenotype	Radiosensitization strategies targeting S100A9	(63)
Neurovascular remodeling	VEGF/VEGFR2; CD146^+^ cancer stem-like cells	Drive angiogenesis and tumor–vascular interaction	Anti-angiogenic therapies; targeting CD146 axis	(64)
Vascular immune regulation	CD276 (B7-H3) expression in endothelial cells	Suppresses T-cell infiltration and immune activation	Immune checkpoint targeting beyond PD-1/PD-L1	(36)
Metabolic–immune coupling	CD39–AMPK signaling; extracellular ATP depletion	Impairs T-cell differentiation and promotes immune evasion	Targeting CD39 or metabolic pathways	(68)
Metabolic adaptation	OXPHOS upregulation; PHGDH-mediated serine synthesis	Supports tumor survival in brain microenvironment	Metabolic inhibitors (e.g., OXPHOS targeting)	(22, 23)
Immune suppression niche	Tregs, M2 macrophages, dysfunctional DCs	Establish “immune-cold” microenvironment	Immunotherapy combination strategies	(33, 70)
mLV dysfunction	Impaired lymphatic drainage and antigen presentation	Reduces immune surveillance and therapy response	Targeting lymphatic–immune axis; enhancing CNS immunity	(44)

DCs, dendritic cells; OXPHOS, oxidative phosphorylation; PHGDH, 3-phosphoglycerate dehydrogenase; RAGE, receptor for advanced glycation end products; STING, stimulator of interferon genes; Tregs, regulatory T cells; Meningeal lymphatic system (mLV).

### 4.1 Astrocyte-mediated tumor colonization and niche formation

During initial colonization, astrocytes serve as primary gatekeepers of the metastatic niche. Tumor–astrocyte interactions are associated with intercellular communication through gap junctions, which facilitate the transfer of signaling molecules such as cGAMP and contribute to tumor progression and therapy resistance (59). In parallel, STAT3^+^ reactive astrocytes are implicated in modulating immune cell activity, including macrophage expansion and CD8^+^ T-cell suppression, thereby reinforcing an immunosuppressive TME (60). To investigate these interactions, astrogliogenesis was induced via PDGF supplementation in advanced models such as MBCCOs, yielding astrocytes that retained neurogenic potential while responding to inflammatory stimuli like TNF-α (61). Mechanistically, tumor cells establish Connexin 43-mediated gap junctions with astrocytes to facilitate pro-proliferative and invasive signaling. Furthermore, lung-specific factors, such as X protein, are implicated in accelerating tumor migration and invasive capacity within this co-culture framework (48).

Crucially, astrocyte–tumor crosstalk extends beyond structural scaffolding to the co-option of neurodevelopmental programs. Organoid co-culture models reveal that SCLC cells recruit reactive astrocytes to establish a supportive niche by hijacking reciprocal neuron–astrocyte developmental pathways. Mechanistically, tumor-derived Reelin mediates astrocyte recruitment, while astrocyte-secreted SERPINE1 reciprocally promotes tumor fitness and expansion (62). Furthermore, this astrocyte-driven niche functions as a refuge for therapeutic resistance. Astrocyte-derived cytokines trigger the S100A9–RAGE–NF-κB–JunB axis in metastatic cells, conferring resistance to whole-brain radiotherapy (WBRT) and promoting cancer stem-like properties (63). Notably, the pharmacological targeting of S100A9 restored radiosensitivity, positioning S100A9 as a dual-purpose predictive biomarker and therapeutic vulnerability in LCBM. In summary, astrocytes function as multifunctional orchestrators of the LCBM niche, transitioning from physical gatekeepers to active biochemical facilitators. These findings, validated in advanced organoid systems, identify the astrocyte–tumor interface as a prime target for overcoming both metastatic progression and treatment failure.

### 4.2 Neurovascular remodeling in structural and immune regulation

Beyond astrocyte-driven colonization, organoid systems further reveal that the neurovascular unit functions as a central regulatory hub integrating vascular remodeling with immune modulation. The interrogation of endothelial and mural cells in brain metastasis reveals key immune-regulatory mechanisms. Notably, vascular-associated immune checkpoint molecules, such as CD276, are significantly upregulated, thereby limiting cytotoxic T-cell infiltration and representing promising therapeutic targets (36). Microfluidic organoid-on-a-chip platforms provide a dynamic and physiologically relevant framework to model tumor–vascular crosstalk in LCBM. Within biomimetic brain metastasis systems, the integration of endothelial cells, astrocytes, and tumor cells enables real-time interrogation of angiogenic processes in a spatially organized neurovascular niche. These organoid-based models demonstrated that patient-derived CD146^+^ cancer stem-like cells actively drove angiogenesis in NSCLC brain metastases through the activation of the VEGF/VEGFR2 signaling axis. Mechanistically, astrocyte-derived GAS6 induced AXL signaling, leading to the upregulation of CD146 and enhanced tumor vascularization, thereby establishing a neuro–tumor–vascular regulatory axis within the metastatic niche (64).

Importantly, neurovascular remodeling extends beyond canonical angiogenesis to encompass pathological processes such as vasculogenic mimicry. Notch signaling-mediated crosstalk among oligodendrocytes, endothelial cells, and microglia drives vasculogenic mimicry in NSCLC-derived BMs (65). However, oligodendrocytes remain underrepresented in cerebral organoids and typically exhibit immature phenotypes even after prolonged culture (6–8 months), although optimized differentiation protocols can generate mature myelinating oligodendrocytes within approximately 5 months (46). Recent studies have also identified a distinct immune population, choroid plexus mast cells, which accumulate within the choroid plexus and disrupt epithelial ciliary function via the tryptase–PAR2–FoxJ1 signaling axis. This disruption leads to increased cerebrospinal fluid production, highlighting a previously underappreciated role of immune cells in driving tumor-associated hydrocephalus (66).

The integration of iPSC-derived microglia-like cells (iMicro) into brain organoids has further elucidated microglia-mediated metabolic signaling within the neurovascular niche. Specifically, PLIN2^+^ lipid droplets in microglia export cholesterol to neural progenitor cells, thereby orchestrating axonogenesis and lineage differentiation (67). This lipid-dependent crosstalk highlights the capacity of organoid assembloids to recapitulate complex homeostatic interactions, providing a sophisticated framework to investigate how metastatic cells may co-opt these developmental pathways to facilitate brain niche colonization.

### 4.3 Metabolic-immune interactions in brain metastasis

Building on the metabolic adaptations described above, organoid-based studies further reveal that metabolic reprogramming is not merely a tumor-intrinsic survival strategy but also a regulator of immune dysfunction in LCBM. In this context, metabolic–immune coupling provides a mechanistic link between brain-specific metabolic adaptation and the formation of an immune-cold metastatic niche. Organoid-based studies further demonstrate that metabolic reprogramming is tightly coupled with immune dysfunction, forming a core mechanism underlying immune evasion in LCBM. Tumor-derived CD39 drove metabolic reprogramming and dysfunctional differentiation of CD4^+^ T cells in NSCLC. Mechanistically, CD39-containing extracellular vesicles released by tumor cells depleted extracellular ATP and activated AMPK signaling in T cells, leading to impaired effector differentiation. These findings, validated in PDOs, highlight a key metabolic-immune axis that contributes to immune evasion and may serve as a potential therapeutic target (68). Beyond cellular recruitment, the metabolic reprogramming of the brain niche serves as a fundamental driver of immune evasion. Duan et al. showed that the hyperactivation of mitochondrial OXPHOS in tumor cells was intrinsically coupled with an immunosuppressed microenvironment (69). This metabolic-immune crosstalk suggests that the brain-specific metabolic shift not only fuels tumor progression but also actively orchestrates an “immune-cold” phenotype, providing a mechanistic basis for the limited efficacy of immune checkpoint blockade monotherapy in LCBM.

Dynamic cerebrospinal fluid profiling of NSCLC patients with BMs identified CD4^+^PDCD1^+^CXCR6^+^ T cells as a positive predictive subset for intracranial response to immune checkpoint inhibitors, functioning through reciprocal interactions with classical DCs to enhance antigen presentation and inflammatory activation (70). In primary lung adenocarcinoma, the NFATc2/galectin-9 axis was shown to support tumor-initiating cell properties and drive immunosuppression via Treg polarization, suggesting that similar mechanisms may operate in the brain metastatic microenvironment (71).

Although organoid-based studies have begun to reveal astrocyte-mediated colonization, vascular immune regulation, and metabolic–immune coupling in LCBM, these mechanisms have not yet been uniformly validated across large patient-derived cohorts. In particular, several pathways identified in organoid or co-culture systems may represent context-dependent interactions rather than universal drivers of brain metastasis. Therefore, the translational significance of these mechanisms depends on whether they can be functionally linked to therapeutic vulnerability, biomarker stratification, and clinically relevant treatment responses.

## 5 Organoid-based platforms for precision immuno-oncology in LCBM

**Figure 4:**
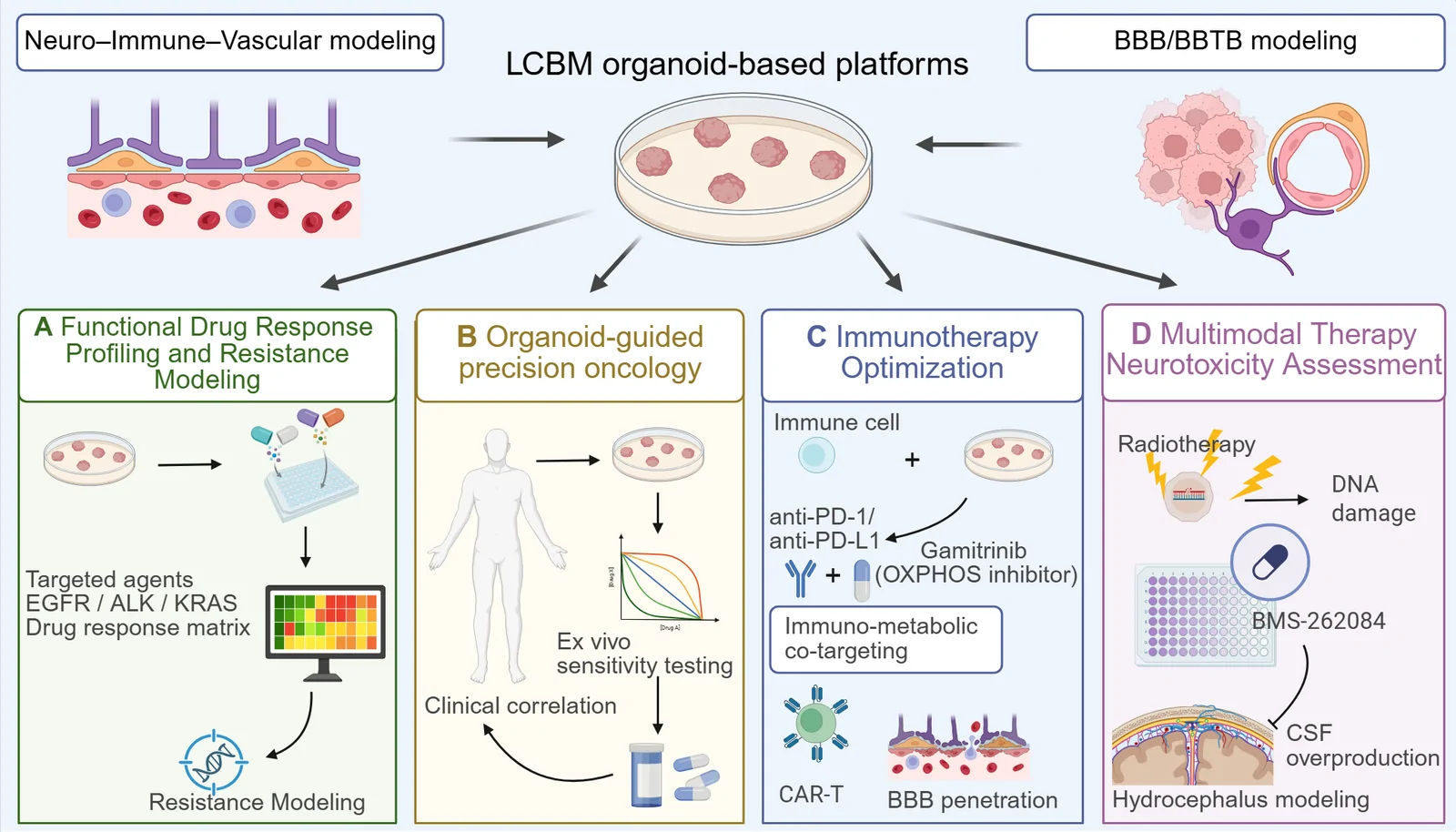
Organoids as platforms for translational therapeutic evaluation and personalized medicine in LCBM. This schematic shows how LCBM organoid-based platforms connect disease modeling with translational therapeutic evaluation. Patient-derived organoids can be combined with BBB/BBTB models, immune cells, and brain microenvironmental components to support drug response profiling, resistance modeling, organoid-guided precision oncology, immunotherapy optimization, and multimodal treatment evaluation. These systems allow assessment of targeted therapies, immune checkpoint blockade, chimeric antigen receptor T-cell (CAR-T) therapy, immunometabolic targeting, radiotherapy response, neurotoxicity, CSF dynamics, and hydrocephalus-related mechanisms, thereby supporting personalized treatment strategies for LCBM. Created with BioRender.com.

Building upon the mechanistic insights described in the previous sections, organoid systems serve as powerful translational platforms that bridge neuro–immune–vascular interactions with precision oncology applications in LCBM (**Figure 4**). Their key applications, including drug response profiling, immunotherapy evaluation, and multimodal treatment modeling, are summarized in **Table 3**.

**Table 3:** Organoid-based therapeutic applications in LCBM.

Application area	Organoid model	Key findings	Clinical relevance	References
Targeted therapy screening	PDOs	Predict sensitivity to *EGFR*, *ALK*, *KRAS*-targeted therapies	Personalized therapy selection	(49, 80)
Drug resistance modeling	PDOs	Identify resistance mutations (e.g., *EGFR* C797S) and alternative strategies	Overcoming therapeutic resistance	(79)
Immunotherapy evaluation	Immune-integrated organoids	Recapitulate tumor–immune interactions; enable assessment of CAR-T cell infiltration, immune checkpoint responses, and T-cell cytotoxicity	Optimization of immunotherapy strategies	(91)
Immuno-metabolic targeting	PDOs, co-culture systems	OXPHOS inhibition enhances anti–PD-1 efficacy	Combination therapy development	(69)
BBB penetration testing	BBB-on-chip/vascularized organoids	Evaluate drug permeability and CNS delivery	Selection of brain-penetrant therapies	(58)
Radiotherapy response	Tumor organoids/cerebral organoids	Reveal heterogeneous radiosensitivity and DNA damage response	Personalized radiotherapy planning	(97)
Neurotoxicity assessment	Cerebral organoids	Evaluate treatment-induced CNS toxicity	Safer therapeutic design	(98)
Precision oncology guidance	PDO-based platforms	Functional drug testing correlates with patient outcomes	Real-time clinical decision-making	(66)

*ALK*, anaplastic lymphoma kinase; BBB, blood–brain barrier; CAR-T, chimeric antigen receptor T-cell; CNS, central nervous system; *EGFR*, epidermal growth factor receptor; *KRAS*, Kirsten rat sarcoma viral oncogene homolog; OXPHOS, oxidative phosphorylation; PDOs, patient-derived organoids.

### 5.1 Functional drug response profiling and resistance modeling in LCBM

The molecular landscape of LCBM critically shapes therapeutic decision-making. Current clinical strategies largely rely on targeted therapies for patients harboring oncogenic driver mutations. Notably, third-generation inhibitors such as osimertinib (*EGFR*) and lorlatinib (*ALK*), alongside emerging therapies targeting *MET*, *ROS1*, *BRAF* V600E, and *KRAS* G12C alterations, have demonstrated significant intracranial efficacy. However, the translational gap remains substantial, as conventional preclinical platforms, namely immortalized cell lines and patient-derived xenografts (PDXs), often lack the fidelity required to accurately predict clinical trajectories, thereby stalling the advancement of personalized medicine (72–77).

LCBM organoids provide a physiologically relevant platform that preserves tumor heterogeneity and genetic features, enabling the functional assessment of drug efficacy and resistance. Preliminary clinical studies using patient-derived tumor spheroids (PDTS) integrated with decellularized extracellular matrix demonstrated the feasibility of generating functional drug sensitivity data within a clinically actionable timeframe, with predictive accuracy reaching approximately 73% in selected cohorts (78).

Beyond routine screening, organoid platforms serve as robust systems for validating next-generation targeted therapies. For instance, PDO-based investigations identified BI-4732 as a potent inhibitor of osimertinib-resistant *EGFR* mutations, specifically the C797S variant. BI-4732 demonstrated superior intracranial activity and BBB penetration, exhibiting synergistic effects when combined with osimertinib to overcome localized therapeutic resistance (79). Furthermore, Kim et al. utilized PDOs to establish the preclinical efficacy of poziotinib and pralsetinib for NSCLC harboring *ERBB2* exon 20 insertions and *RET* fusions, respectively, highlighting the role of organoids in personalizing strategies for rare oncogenic alterations (49). Similarly, in LCBM models driven by *KRAS* G12C mutation and *CDKN2A* loss, the combination of adagrasib and the brain-penetrant CDK4/6 inhibitor abemaciclib significantly extended survival, providing a functional rationale for multi-target combinatorial approaches in aggressive metastatic subtypes (80).

Importantly, the integration of metastatic tumor cells with cerebral organoids enables simultaneous evaluation of therapeutic efficacy, CNS penetration, and neurotoxicity. In a cerebral organoid model of melanoma brain metastasis, the XPO1 inhibitor selinexor was identified as an effective agent that suppressed tumor growth while exhibiting minimal neurotoxicity (81). Although these findings provide a useful proof of concept for the parallel assessment of antitumor activity and neural toxicity in brain metastasis organoid systems, further validation in LCBM-specific models is required. Collectively, these findings establish organoids as mechanism-guided platforms linking molecular alterations to functional therapeutic responses.

### 5.2 Organoid-guided precision oncology and clinical correlation in LCBM

While organoid systems enable functional drug response profiling, their greater clinical value lies in guiding patient-specific therapeutic decision-making. By preserving tumor heterogeneity and microenvironmental features, PDOs provide a functional framework that complements genomic profiling and refines personalized treatment strategies.

Qin et al. demonstrated that organoid-based sensitivity testing effectively tailored therapies for complex cases. Specifically, it identified potent responses to a triplet regimen (pemetrexed, carboplatin, and osimertinib) in *EGFR*-mutant tumors, or selectively uncovered brigatinib responsiveness in patients with refractory *ALK* fusion variants, leading to durable clinical remission (82). Furthermore, organoids can reveal therapeutic vulnerabilities that bypass conventional genomic predictions. Pan et al. established a PDO model from a patient with *EGFR* wild-type lung adenocarcinoma (LUAD) which, despite the absence of canonical mutations, exhibited remarkable sensitivity to gefitinib monotherapy. This finding was subsequently validated by the patient’s sustained clinical response, highlighting the capacity of PDOs to capture non-canonical drug sensitivities that genomic sequencing alone might overlook (83). Although emerging clinical studies suggest the potential utility of PDO-guided functional assays in refining treatment selection for LCBM, the current evidence remains limited, as most reports are derived from case studies and retrospective analyses, and prospective validation is still required.

Beyond individual case studies, Wang et al. discovered that PDOs effectively predict chemotherapy and targeted therapy resistance, with drug sensitivity tests accurately reflecting clinical responses and aiding in the development of personalized treatment strategies for various subtypes of advanced lung cancer (84).

Collectively, these clinical correlations underscore the value of organoids as functional platforms that enhance therapeutic stratification and improve the precision of clinical decision-making. Importantly, organoid-based approaches shift precision oncology from mutation-guided therapy toward functionally validated therapeutic strategies, offering a promising avenue for improving clinical outcomes in LCBM. Nevertheless, current patient–organoid clinical correlations remain limited by small cohort sizes, tumor sampling bias, and the frequent absence of stromal and immune compartments in conventional PDO systems (85).

### 5.3 Immuno-functional interrogation and immunotherapy optimization

Organoid models now provide a dynamic platform to test inhibitors against specific targets in a reconstructed TME. The TME of LCBM is characterized by complex interactions between immune cells, stromal components, and tumor-derived factors, rendering immunotherapy a critical yet challenging therapeutic strategy. ICIs, particularly those targeting the PD-1/PD-L1 axis, have emerged as central treatments for LCBM (86). A recent large-scale meta-analysis demonstrated that ICIs conferred meaningful survival benefits in these patients, although durable responses remain limited (87). Importantly, therapeutic efficacy is strongly influenced by tumor genomic context: patients harboring *EGFR* mutations generally exhibited poor responses to ICI monotherapy (88), whereas *KRAS*-mutant tumors were associated with an inflammatory TME and enhanced immunogenicity, leading to improved responsiveness to PD-1/PD-L1 blockade (89).

Conventional models fail to fully recapitulate the complexity of tumor–immune interactions within the brain metastatic niche. In contrast, organoid platforms reconstruct multicellular tumor architecture and preserve key components of the microenvironment, enabling functional interrogation of immune responses (90). In this setting, organoid-based systems have been applied to optimize immunotherapeutic strategies, including enhancing CAR-T cell trafficking across BBB-like structures and improving intratumoral infiltration (91).

Building on the immuno-metabolic mechanisms described above, organoid platforms provide a functional system for testing whether metabolic vulnerabilities can be therapeutically exploited to improve immunotherapy responses. Functional studies using PDOs demonstrated that inhibiting OXPHOS with gamitrinib induced tumor apoptosis; notably, its combination with anti-PD-1 therapy exerted a potent synergistic effect and significantly prolonged survival in preclinical models (69). This synergy highlights the value of organoids in validating immuno-metabolic co-targeting as a viable strategy to overcome the cold metastatic microenvironment.

Despite the relatively limited number of studies directly applying brain metastasis organoids in LCBM, accumulating evidence from other tumor types highlights the robustness of organoid platforms in immunotherapy research. In diverse malignancies, organoid systems have been utilized to reconstruct tumor–immune interactions and assess immune checkpoint responses (92–95). Given the unique immunologically “cold” or sequestered status of the brain, LCBM organoid models represent an indispensable tool for deciphering immune evasion and optimizing personalized immunotherapy.

### 5.4 Multi-modal therapy modeling and neurotoxicity assessment

Radiotherapy remains a cornerstone in the management of LCBM and is increasingly combined with systemic therapies to improve intracranial control. A phase 2 trial demonstrated that the integration of brain radiotherapy with immune checkpoint inhibition and chemotherapy achieved encouraging intracranial efficacy in NSCLC brain metastases, highlighting the potential of radio-immunotherapy combinations to overcome resistance (96). Organoids can serve as the ideal platform to optimize the timing and dosing of such complex multimodal regimens, predicting which patients will experience synergistic therapeutic effects versus treatment-limiting toxicity.

Beyond tumor response, organoid systems can be leveraged to investigate treatment-associated complications and CNS-specific effects. For example, patient-derived tumoroids were employed to assess dose-dependent treatment effects, with DNA damage and cell death quantified via cell viability assays and immunofluorescence staining. These studies revealed heterogeneous radiosensitivity, with radiation-resistant organoids exhibiting reduced DNA damage and enhanced survival (97).

Cerebral organoids have also been applied to model radiation-induced brain injury, demonstrating their utility in evaluating therapy-associated neurotoxicity in a physiologically relevant context (98). This approach has been further extended to investigate complications associated with LCBM. For example, in tumor-associated hydrocephalus, the small-molecule tryptase inhibitor BMS-262084 was shown to effectively cross the BBB and suppress cerebrospinal fluid overproduction by preserving choroid plexus epithelial cilia integrity. This finding not only provides a novel immunomodulatory therapeutic strategy but also underscores the advantages of advanced organoid systems in evaluating CNS drug penetration (66).

Collectively, these findings position organoid platforms as an essential nexus for precision immuno-oncology, enabling the recapitulation of complex tumor-immune interactions and the optimization of combinatorial immunotherapy in LCBM. While the application of these models in radiotherapy and dynamic immune trafficking remains a burgeoning frontier, their capacity to functionally validate immuno-metabolic targets and assess immune-mediated neurotoxicity provides a critical translational bridge toward achieving truly personalized therapeutic strategies for LCBM.

## 6 Discussion

Despite the rapid advancement of organoid-based platforms in modeling LCBM, several critical challenges continue to limit their translational potential. A primary obstacle lies in the lack of standardization across culture systems (99). Variability in tissue sources, culture conditions, extracellular matrix composition, passage number and differentiation protocols across laboratories results in inconsistencies that hinder experimental reproducibility. Therefore, future studies should establish standardized operating procedures, quality-control criteria, and benchmarking frameworks. In addition, the incomplete recapitulation of the intricate neuro–immune–vascular niche remains a major limitation. Although current organoid models increasingly incorporate multiple cell types, they still fail to fully recapitulate the brain metastatic niche owing to incomplete representation of vascular, immune, and glial compartments and insufficient spatiotemporal dynamics of metastatic progression. Integration of perfusable microfluidic systems capable of reproducing blood flow, shear stress, transendothelial migration, and BBB remodeling would substantially improve the physiological relevance of these models while enabling more accurate evaluation of drug penetration, immune-cell trafficking, and anti-angiogenic resistance. At the same time, a key challenge for the field is to avoid equating increasing model complexity with increasing biological relevance. The most useful LCBM organoid platforms will likely be those designed to answer clearly defined questions, such as BBB penetration, immune-cell trafficking, astrocyte-mediated resistance, or immuno-metabolic vulnerability. Therefore, future studies should match model complexity to the biological or therapeutic question being addressed, rather than pursuing comprehensive reconstruction of the entire metastatic niche in every system.

Beyond the vascular niche, incorporation of meningeal lymphatic-like structures represents an important but underexplored frontier. Emerging evidence highlights the missing role of meningeal lymphatics (mLVs), which are now recognized as critical conduits for immune surveillance and therapeutic drainage. A key future direction is the integration of meningeal lymphatic vessels into next-generation LCBM organoid and organ-on-a-chip systems. Emerging bioengineering approaches, including the incorporation of lymphatic endothelial cells and the construction of meninges-mimetic microfluidic compartments, may enable partial reconstruction of mLV-like structures *in vitro* (100). Such systems could help investigate cerebrospinal fluid dynamics, immune-cell egress to cervical lymph nodes, and lymphatic-mediated immunoregulation, thereby narrowing the gap between organoid models and the *in vivo* metastatic microenvironment.

Therapeutic resistance remains a major challenge in LCBM, highlighting the need for more scalable organoid-based screening platforms. Current small-molecule screens in organoids are generally restricted to modest therapeutic panels, but ultra-high-throughput miniaturized culture systems could expand screening capacity to larger compound libraries (101). Moreover, advances that enable large-scale expansion of organoid biomass would further increase the feasibility and scale of both genetic and pharmacological screens (102). These platforms could also be used for perturbation-based screening, including CRISPR-based target discovery, cytokine blockade, metabolic inhibition, and rational drug-combination testing.

The application of organoid systems to radiotherapy research remains relatively underdeveloped. Beyond evaluating intrinsic radiosensitivity, these platforms offer opportunities to investigate radiation-induced remodeling of the tumor microenvironment. Organoid-based assembloids may enable modeling of the interplay between ionizing radiation and immune responses, thereby facilitating the identification of optimal therapeutic windows for radio-immunotherapy combinations. Furthermore, region-specific brain organoids provide a promising framework for assessing neurotoxicity and distinguishing radiation necrosis from tumor progression (103).

The integration of autologous immune compartments stands as the critical frontier in refining precision immuno-oncology for LCBM. Given the profound inter-patient heterogeneity observed with modalities like B7-H3-targeted CAR-T cells and oncolytic viruses, chimeroid platforms provide a human-centric scaffold that transcends the cross-species limitations of murine models (104, 105). By enabling the functional validation of immune-cell trafficking and cytotoxicity within a specific spatial context, these next-generation organoids offer a scalable pathway to mitigate therapeutic resistance and optimize clinical stratification. However, their application in LCBM remains at an early stage and requires further validation.

Perhaps the most revolutionary potential applications of organoids for oncology reside in therapeutic prediction. Numerous studies have demonstrated the feasibility of organoid-based functional testing for patient-specific drug-response assessment; however, prospective validation in independent cohorts is still required. To support clinical implementation, organoid-guided assays should provide results within clinically actionable timeframes, demonstrate improved patient outcomes, and potentially be extended to modalities such as immunotherapy. To move toward clinical implementation, establishing LCBM organoid biobanks linked to clinical annotation, neuroimaging, treatment history, genomic profiles, and longitudinal outcomes would provide an essential infrastructure for validating organoid-based prediction models.

Rather than replacing animal models or clinical studies, next-generation LCBM organoids should be positioned as complementary human-relevant platforms. With the improved reconstruction of the neuro–immune–vascular–lymphatic niche, organoid-based systems may evolve from descriptive disease models into clinically actionable tools for improving the management of patients with LCBM.

## References

[1] Nayak L Lee EQ Wen PY . Epidemiology of brain metastases. Curr Oncol Rep. (2012) 14:48–54. doi: 10.1007/s11912-011-0203-y 22012633

[2] Sung H Ferlay J Siegel RL Laversanne M Soerjomataram I Jemal A . Global cancer statistics 2020: Globocan estimates of incidence and mortality worldwide for 36 cancers in 185 countries. CA: A Cancer J For Clin. (2021) 71:209–49. doi: 10.3322/caac.21660 33538338

[3] Ernani V Stinchcombe TE . Management of brain metastases in non-small-cell lung cancer. J Oncol Pract. (2019) 15:563–70. doi: 10.1200/jop.19.00357 31715122 PMC7098835

[4] Zhu Y Cui Y Zheng X Zhao Y Sun G . Small-cell lung cancer brain metastasis: From molecular mechanisms to diagnosis and treatment. Biochim Biophys Acta Mol Basis Dis. (2022) 1868:166557. doi: 10.1016/j.bbadis.2022.166557 36162624

[5] Lu Y Huang Y Zhu C Li Z Zhang B Sheng H . Cancer brain metastasis: Molecular mechanisms and therapeutic strategies. Mol BioMed. (2025) 6:12. doi: 10.1186/s43556-025-00251-0 39998776 PMC11861501

[6] Ma SC Bai X Guo XJ Liu L Xiao LS Lin Y . Organ-specific metastatic landscape dissects pd-(l)1 blockade efficacy in advanced non-small cell lung cancer: Applicability from clinical trials to real-world practice. BMC Med. (2022) 20:120. doi: 10.1186/s12916-022-02315-2 35410334 PMC9004108

[7] Solomon BJ Bauer TM Mok TSK Liu G Mazieres J de Marinis F . Efficacy and safety of first-line lorlatinib versus crizotinib in patients with advanced, alk-positive non-small-cell lung cancer: Updated analysis of data from the phase 3, randomised, open-label crown study. Lancet Respir Med. (2023) 11:354–66. doi: 10.1016/s2213-2600(22)00437-4 36535300

[8] Li H Chen K Gong L Qin J Jin Y Zhou R . High-dose aumolertinib for untreated egfr-variant non-small cell lung cancer with brain metastases: The achieve phase 2 nonrandomized clinical trial. JAMA Oncol. (2025) 11:900–8. doi: 10.1001/jamaoncol.2025.1779 40569623 PMC12203398

[9] Zhou Q Yu Y Xing L Cheng Y Wang Y Pan Y . First-line zorifertinib for egfr-mutant non-small cell lung cancer with central nervous system metastases: The phase 3 everest trial. Med. (2025) 6:100513. doi: 10.1016/j.medj.2024.09.002 39389055

[10] Junttila MR Repellin CE Salaniwal S Warne R Lee Y Kim H . Enozertinib is a selective, brain-penetrant egfr inhibitor for treating non-small cell lung cancers with egfr exon 20 and atypical mutations. Cancer Res. (2026) 86:759–72. doi: 10.1158/0008-5472.Can-25-3502 41196054

[11] Wang S Uriel M Cheng H . Lung cancer with brain metastasis-treatment strategies and molecular characteristics. J Clin Med. (2024) 13. doi: 10.3390/jcm13237371 39685828 PMC11641837

[12] Alsabbagh R Ahmed M Alqudah MAY Hamoudi R Harati R . Insights into the molecular mechanisms mediating extravasation in brain metastasis of breast cancer, melanoma, and lung cancer. Cancers. (2023) 15. doi: 10.3390/cancers15082258 37190188 PMC10137309

[13] Liu W Powell CA Wang Q . Tumor microenvironment in lung cancer-derived brain metastasis. Chin Med J. (2022) 135:1781–91. doi: 10.1097/cm9.0000000000002127 35838548 PMC9521756

[14] Kim MS Lee J Lee JE An JH Heo JY Yeo MK . Brain metastasis from non-small cell lung cancer: Crosstalk between cancer cells and tumor microenvironment components. Exp Mol Med. (2025) 57:2749–62. doi: 10.1038/s12276-025-01604-z 41429896 PMC12800198

[15] Invrea F Rovito R Torchiaro E Petti C Isella C Medico E . Patient-derived xenografts (pdxs) as model systems for human cancer. Curr Opin Biotechnol. (2020) 63:151–6. doi: 10.1016/j.copbio.2020.01.003 32070860

[16] Kang HN Choi JW Shim HS Kim J Kim DJ Lee CY . Establishment of a platform of non-small-cell lung cancer patient-derived xenografts with clinical and genomic annotation. Lung Cancer. (2018) 124:168–78. doi: 10.1016/j.lungcan.2018.08.008 30268457

[17] Gonzales-Aloy E Ahmed-Cox A Tsoli M Ziegler DS Kavallaris M . From cells to organoids: The evolution of blood-brain barrier technology for modelling drug delivery in brain cancer. Adv Drug Delivery Rev. (2023) 196:114777. doi: 10.1016/j.addr.2023.114777 36931346

[18] Peinado H Zhang H Matei IR Costa-Silva B Hoshino A Rodrigues G . Pre-metastatic niches: Organ-specific homes for metastases. Nat Rev Cancer. (2017) 17:302–17. doi: 10.1038/nrc.2017.6 28303905

[19] Frezzetti D Gallo M Roma C D'Alessio A Maiello MR Bevilacqua S . Vascular endothelial growth factor a regulates the secretion of different angiogenic factors in lung cancer cells. J Cell Physiol. (2016) 231:1514–21. doi: 10.1002/jcp.25243 26542886

[20] Ciminera AK Jandial R Termini J . Metabolic advantages and vulnerabilities in brain metastases. Clin Exp Metastasis. (2017) 34:401–10. doi: 10.1007/s10585-017-9864-8 29063238 PMC5712254

[21] Pinto M Violante S Cascão R Faria CC . Unlocking the role of metabolic pathways in brain metastatic disease. Cells. (2025) 14. doi: 10.3390/cells14100707 40422210 PMC12109720

[22] Fukumura K Malgulwar PB Fischer GM Hu X Mao X Song X . Multi-omic molecular profiling reveals potentially targetable abnormalities shared across multiple histologies of brain metastasis. Acta Neuropathol. (2021) 141:303–21. doi: 10.1007/s00401-020-02256-1 33394124 PMC7852029

[23] Ngo B Kim E Osorio-Vasquez V Doll S Bustraan S Liang RJ . Limited environmental serine and glycine confer brain metastasis sensitivity to phgdh inhibition. Cancer Discov. (2020) 10:1352–73. doi: 10.1158/2159-8290.Cd-19-1228 32571778 PMC7483776

[24] Xie M Qin H Liu L Wu J Zhao Z Zhao Y . Gaba regulates metabolic reprogramming to mediate the development of brain metastasis in non-small cell lung cancer. J Exp Clin Cancer Res. (2025) 44:61. doi: 10.1186/s13046-025-03315-9 39972344 PMC11837350

[25] Chen B Kiang KM Liu F Li C Li X Weiwei C . Neutrophil extracellular trap reprograms cancer metabolism to form a metastatic niche promoting non-small cell lung cancer brain metastasis. Adv Sci. (2026) 13:e08478. doi: 10.1002/advs.202508478 41250997 PMC12850224

[26] Parida PK Malladi S . Metabolic adaptations of brain metastasis. Nat Rev Cancer. (2025) 25:723–39. doi: 10.1038/s41568-025-00848-1 40707738

[27] Quail DF Joyce JA . The microenvironmental landscape of brain tumors. Cancer Cell. (2017) 31:326–41. doi: 10.1016/j.ccell.2017.02.009 28292436 PMC5424263

[28] Xing X Zhong J Biermann J Duan H Zhang X Shi Y . Pan-cancer human brain metastases atlas at single-cell resolution. Cancer Cell. (2025) 43:1242–60.e9. doi: 10.1016/j.ccell.2025.03.025 40215980 PMC12323703

[29] Vilariño N Bruna J Bosch-Barrera J Valiente M Nadal E . Immunotherapy in nsclc patients with brain metastases. Understanding brain tumor microenvironment and dissecting outcomes from immune checkpoint blockade in the clinic. Cancer Treat Rev. (2020) 89:102067. doi: 10.1016/j.ctrv.2020.102067 32682248

[30] Friebel E Kapolou K Unger S Núñez NG Utz S Rushing EJ . Single-cell mapping of human brain cancer reveals tumor-specific instruction of tissue-invading leukocytes. Cell. (2020) 181:1626–42.e20. doi: 10.1016/j.cell.2020.04.055 32470397

[31] Guldner IH Wang Q Yang L Golomb SM Zhao Z Lopez JA . Cns-native myeloid cells drive immune suppression in the brain metastatic niche through cxcl10. Cell. (2020) 183:1234–48.e25. doi: 10.1016/j.cell.2020.09.064 33113353 PMC7704908

[32] Badillo-Godinez O Niemi J Helfridsson L Karimi S Ramachandran M Mangukiya HB . Brain tumors induce immunoregulatory dendritic cells in draining lymph nodes that can be targeted by ox40 agonist treatment. J Immunother Cancer. (2025) 13. doi: 10.1136/jitc-2025-011548 40389372 PMC12090865

[33] Guan C Li X Zeng X Du Z Zhou Z Zhao J . Unraveling the alterations and biomarkers in the tumor microenvironment in lung adenocarcinoma metastases and their indications for therapeutic response and prognosis. Ther Adv Med Oncol. (2025) 17:17588359251403904. doi: 10.1177/17588359251403904 41437936 PMC12719589

[34] Correia AL . Locally sourced: Site-specific immune barriers to metastasis. Nat Rev Immunol. (2023) 23:522–38. doi: 10.1038/s41577-023-00836-2 36750616 PMC9904275

[35] Zhang Q Abdo R Iosef C Kaneko T Cecchini M Han VK . The spatial transcriptomic landscape of non-small cell lung cancer brain metastasis. Nat Commun. (2022) 13:5983. doi: 10.1038/s41467-022-33365-y 36216799 PMC9551067

[36] Bejarano L Kauzlaric A Lamprou E Lourenco J Fournier N Ballabio M . Interrogation of endothelial and mural cells in brain metastasis reveals key immune-regulatory mechanisms. Cancer Cell. (2024) 42:378–95.e10. doi: 10.1016/j.ccell.2023.12.018 38242126

[37] Xiao Y Hu F Chi Q . Single-cell rna sequencing and spatial transcriptome reveal potential molecular mechanisms of lung cancer brain metastasis. Int Immunopharmacol. (2024) 140:112804. doi: 10.1016/j.intimp.2024.112804 39079345

[38] Arvanitis CD Ferraro GB Jain RK . The blood-brain barrier and blood-tumour barrier in brain tumours and metastases. Nat Rev Cancer. (2020) 20:26–41. doi: 10.1038/s41568-019-0205-x 31601988 PMC8246629

[39] Geissler M Jia W Kiraz EN Kulacz I Liu X Rombach A . The brain pre-metastatic niche: Biological and technical advancements. Int J Mol Sci. (2023) 24. doi: 10.3390/ijms241210055 37373202 PMC10298140

[40] Agnihotri TG Salave S Shinde T Srikanth I Gyanani V Haley JC . Understanding the role of endothelial cells in brain tumor formation and metastasis: A proposition to be explored for better therapy. J Natl Cancer Cent. (2023) 3:222–35. doi: 10.1016/j.jncc.2023.08.001 39035200 PMC11256543

[41] Marchetti L Engelhardt B . Immune cell trafficking across the blood-brain barrier in the absence and presence of neuroinflammation. Vasc Biol. (2020) 2:H1–h18. doi: 10.1530/vb-19-0033 32923970 PMC7439848

[42] Daneman R Zhou L Kebede AA Barres BA . Pericytes are required for blood-brain barrier integrity during embryogenesis. Nature. (2010) 468:562–6. doi: 10.1038/nature09513 20944625 PMC3241506

[43] Cucullo L Hossain M Puvenna V Marchi N Janigro D . The role of shear stress in blood-brain barrier endothelial physiology. BMC Neurosci. (2011) 12:40. doi: 10.1186/1471-2202-12-40 21569296 PMC3103473

[44] Zhang Y Jin S Guo P Yin Y Hua Y Ding X . Magnetic resonance imaging reveals meningeal lymphatic impairment in lung adenocarcinoma brain metastasis progression. Adv Sci. (2026) 13:e16988. doi: 10.1002/advs.202516988 41538419 PMC13042558

[45] Polak R Zhang ET Kuo CJ . Cancer organoids 2.0: Modelling the complexity of the tumour immune microenvironment. Nat Rev Cancer. (2024) 24:523–39. doi: 10.1038/s41568-024-00706-6 38977835

[46] Lancaster MA Renner M Martin CA Wenzel D Bicknell LS Hurles ME . Cerebral organoids model human brain development and microcephaly. Nature. (2013) 501:373–9. doi: 10.1038/nature12517 23995685 PMC3817409

[47] Endo H Okami J Okuyama H Kumagai T Uchida J Kondo J . Spheroid culture of primary lung cancer cells with neuregulin 1/her3 pathway activation. J Thorac Oncol. (2013) 8:131–9. doi: 10.1097/JTO.0b013e3182779ccf 23328545

[48] Choe MS Kim JS Yeo HC Bae CM Han HJ Baek K . A simple metastatic brain cancer model using human embryonic stem cell-derived cerebral organoids. FASEB J. (2020) 34:16464–75. doi: 10.1096/fj.202000372R 33099835

[49] Kim SY Kim SM Lim S Lee JY Choi SJ Yang SD . Modeling clinical responses to targeted therapies by patient-derived organoids of advanced lung adenocarcinoma. Clin Cancer Res. (2021) 27:4397–409. doi: 10.1158/1078-0432.Ccr-20-5026 34083237 PMC9401503

[50] Fitzpatrick A Iravani M Mills A Vicente D Alaguthurai T Roxanis I . Genomic profiling and pre-clinical modelling of breast cancer leptomeningeal metastasis reveals acquisition of a lobular-like phenotype. Nat Commun. (2023) 14:7408. doi: 10.1038/s41467-023-43242-x 37973922 PMC10654396

[51] Reed-McBain C Turaga RV Zima SRT Patel J Cunha AWF Mixdorf J . Non-destructive luminescence and pet imaging to monitor tissue microenvironment in microphysiological systems during brain metastasis using dissociated cerebral organoids. Biofabrication. (2025) 17. doi: 10.1088/1758-5090/ade1fb 40480250

[52] Cakir B Xiang Y Tanaka Y Kural MH Parent M Kang YJ . Engineering of human brain organoids with a functional vascular-like system. Nat Methods. (2019) 16:1169–75. doi: 10.1038/s41592-019-0586-5 31591580 PMC6918722

[53] Yang L Liu Q Zhang X Liu X Zhou B Chen J . DNA of neutrophil extracellular traps promotes cancer metastasis via ccdc25. Nature. (2020) 583:133–8. doi: 10.1038/s41586-020-2394-6 32528174

[54] Peng T Ma X Hua W Wang C Chu Y Sun M . Individualized patient tumor organoids faithfully preserve human brain tumor ecosystems and predict patient response to therapy. Cell Stem Cell. (2025) 32:652–669.e11. doi: 10.1016/j.stem.2025.01.002 39938519

[55] Baumann Z Billy E Scheidmann MC . Advancements in organoid models emulating metastatic niches. Trends Cancer. (2026). doi: 10.1016/j.trecan.2026.02.005 41813475

[56] Dao L You Z Lu L Xu T Sarkar AK Zhu H . Modeling blood-brain barrier formation and cerebral cavernous malformations in human psc-derived organoids. Cell Stem Cell. (2024) 31:818–833.e11. doi: 10.1016/j.stem.2024.04.019 38754427 PMC11162335

[57] Nogueira Pinto H Kok NR Hauger PC Karsten-van Diepen M de Kok M Paauw NJ . Brain pericytes and wnt/β-catenin signaling induce functional blood-brain barrier phenotype in human ipsc-derived model. Small Methods. (2026):e02114. doi: 10.1002/smtd.202502114 41601407

[58] Du Y Wang YR Bao QY Xu XX Xu C Wang S . Personalized vascularized tumor organoid-on-a-chip for tumor metastasis and therapeutic targeting assessment. Adv Mater. (2025) 37:e2412815. doi: 10.1002/adma.202412815 39726096

[59] Chen Q Boire A Jin X Valiente M Er EE Lopez-Soto A . Carcinoma-astrocyte gap junctions promote brain metastasis by cgamp transfer. Nature. (2016) 533:493–8. doi: 10.1038/nature18268 27225120 PMC5021195

[60] Priego N Zhu L Monteiro C Mulders M Wasilewski D Bindeman W . Stat3 labels a subpopulation of reactive astrocytes required for brain metastasis. Nat Med. (2018) 24:1024–35. doi: 10.1038/s41591-018-0044-4 29892069

[61] Wang M Zhang L Novak SW Yu J Gallina IS Xu LL . Morphological diversification and functional maturation of human astrocytes in glia-enriched cortical organoid transplanted in mouse brain. Nat Biotechnol. (2025) 43:52–62. doi: 10.1038/s41587-024-02157-8 38418648 PMC11349933

[62] Qu F Brough SC Michno W Madubata CJ Hartmann GG Puno A . Crosstalk between small-cell lung cancer cells and astrocytes mimics brain development to promote brain metastasis. Nat Cell Biol. (2023) 25:1506–19. doi: 10.1038/s41556-023-01241-6 37783795 PMC11230587

[63] Monteiro C Miarka L Perea-García M Priego N García-Gómez P Álvaro-Espinosa L . Stratification of radiosensitive brain metastases based on an actionable s100a9/rage resistance mechanism. Nat Med. (2022) 28:752–65. doi: 10.1038/s41591-022-01749-8 35411077 PMC9018424

[64] Liu W Duan W Xia S Liu Y Chu H Liang K . Cd146(+) pericyte-like lung cancer brain metastatic stem cells promote tumor angiogenesis through dual regulatory effects on the vegf/vegfr axis. Theranostics. (2026) 16:1905–24. doi: 10.7150/thno.122241 41356185 PMC12680595

[65] Souza VGP Telkar N Lam WL Reis PP . Comprehensive analysis of lung adenocarcinoma and brain metastasis through integrated single-cell transcriptomics. Int J Mol Sci. (2024) 25. doi: 10.3390/ijms25073779 38612588 PMC11012108

[66] Li Y Di C Song S Zhang Y Lu Y Liao J . Choroid plexus mast cells drive tumor-associated hydrocephalus. Cell. (2023) 186:5719–5738.e28. doi: 10.1016/j.cell.2023.11.001 38056463

[67] Park DS Kozaki T Tiwari SK Moreira M Khalilnezhad A Torta F . Ips-cell-derived microglia promote brain organoid maturation via cholesterol transfer. Nature. (2023) 623:397–405. doi: 10.1038/s41586-023-06713-1 37914940

[68] Wang Y Liu M Zhang L Liu X Ji H Wang Y . Cancer cd39 drives metabolic adaption and mal-differentiation of cd4(+) t cells in patients with non-small-cell lung cancer. Cell Death Dis. (2023) 14:804. doi: 10.1038/s41419-023-06336-4 38062068 PMC10703826

[69] Duan H Ren J Wei S Yang Z Li C Wang Z . Integrated analyses of multi-omic data derived from paired primary lung cancer and brain metastasis reveal the metabolic vulnerability as a novel therapeutic target. Genome Med. (2024) 16:138. doi: 10.1186/s13073-024-01410-8 39593114 PMC11590298

[70] Li YS Lai W Yin K Tu HY Li L Lin SH . Cellular dynamics in cerebrospinal fluid unveils the key regulators of intracranial response to immune checkpoint inhibitors in nsclc brain metastases. J Immunother Cancer. (2025) 13. doi: 10.1136/jitc-2025-012071 41314983 PMC12666088

[71] Xiao ZJ Wang SQ Chen JJ Li Y Jiang Y Tin VP . The dual role of the nfatc2/galectin-9 axis in modulating tumor-initiating cell phenotypes and immune suppression in lung adenocarcinoma. Adv Sci. (2024) 11:e2306059. doi: 10.1002/advs.202306059 38528665 PMC11132051

[72] Jänne PA Planchard D Kobayashi K Cheng Y Lee CK Valdiviezo N . Cns efficacy of osimertinib with or without chemotherapy in epidermal growth factor receptor-mutated advanced non-small-cell lung cancer. J Clin Oncol. (2024) 42:808–20. doi: 10.1200/jco.23.02219 38042525 PMC10906563

[73] Solomon BJ Liu G Felip E Mok TSK Soo RA Mazieres J . Lorlatinib versus crizotinib in patients with advanced alk-positive non-small cell lung cancer: 5-year outcomes from the phase iii crown study. J Clin Oncol. (2024) 42:3400–9. doi: 10.1200/jco.24.00581 38819031 PMC11458101

[74] Wolf J Hochmair M Han JY Reguart N Souquet PJ Smit EF . Capmatinib in met exon 14-mutated non-small-cell lung cancer: Final results from the open-label, phase 2 geometry mono-1 trial. Lancet Oncol. (2024) 25:1357–70. doi: 10.1016/s1470-2045(24)00441-8 39362249

[75] Drilon A Camidge DR Lin JJ Kim SW Solomon BJ Dziadziuszko R . Repotrectinib in ros1 fusion-positive non-small-cell lung cancer. N Engl J Med. (2024) 390:118–31. doi: 10.1056/NEJMoa2302299 38197815 PMC11702311

[76] Riely GJ Ahn MJ Clarke JM Dagogo-Jack I Esper R Felip E . Updated efficacy and safety from the phase 2 pharos study of encorafenib plus binimetinib in patients with braf v600e-mutant metastatic nsclc-a brief report. J Thorac Oncol. (2025) 20:1538–47. doi: 10.1016/j.jtho.2025.05.023 40480428 PMC12451588

[77] Dingemans AC Syrigos K Livi L Paulus A Kim SW Chen Y . Intracranial activity of sotorasib vs docetaxel in pretreated kras g12c-mutated advanced non-small cell lung cancer from a global, phase 3, randomized controlled trial. Lung Cancer. (2025) 207:108683. doi: 10.1016/j.lungcan.2025.108683 40774040

[78] Chang CJ Kan KW Shih YY Nan YH Juan YC Tu CH . Preliminary study of utilizing a patient derived tumor spheroid model to augment precision therapy in metastatic brain tumors. Sci Rep. (2024) 14:31888. doi: 10.1038/s41598-024-83409-0 39738713 PMC11685586

[79] Lee EJ Oh SY Lee YW Kim JY Kim MJ Kim TH . Discovery of a novel potent egfr inhibitor against egfr activating mutations and on-target resistance in nsclc. Clin Cancer Res. (2024) 30:1582–94. doi: 10.1158/1078-0432.Ccr-23-2951 38330145

[80] Migliarese C Sadeh Y Torrini C Turna Demir F Nayyar N Yamazawa E . Combination therapy of adagrasib and abemaciclib in non-small cell lung cancer brain metastasis models genomically characterized by kras-g12c and homozygous loss of cdkn2a. Acta Neuropathol Commun. (2025) 13:88. doi: 10.1186/s40478-025-01993-2 40317086 PMC12046717

[81] Krieg K Materna-Reichelt S Naber T RaChad FZ Kauven P Weller A . Cortical organoid-derived models of the melanoma brain metastatic niche enable prioritization of cancer-targeting drugs. Cell Rep Methods. (2025) 5:101236. doi: 10.1016/j.crmeth.2025.101236 41240911 PMC12859518

[82] Qin L Wang S Li L Qin H . Patient-derived organoid facilitating personalized medicine in non-small cell lung cancer: Two case reports. Front Oncol. (2025) 15. doi: 10.3389/fonc.2025.1674897 41114337 PMC12531068

[83] Pan Y Cui H Song Y . Organoid drug screening report for a non-small cell lung cancer patient with egfr gene mutation negativity: A case report and review of the literature. Front Oncol. (2023) 13. doi: 10.3389/fonc.2023.1109274 36874139 PMC9978590

[84] Wang HM Zhang CY Peng KC Chen ZX Su JW Li YF . Using patient-derived organoids to predict locally advanced or metastatic lung cancer tumor response: A real-world study. Cell Rep Med. (2023) 4:100911. doi: 10.1016/j.xcrm.2022.100911 36657446 PMC9975107

[85] Huang Z Zhang Z Zhou C Liu L Huang C . Epithelial-mesenchymal transition: The history, regulatory mechanism, and cancer therapeutic opportunities. MedComm. (2022) 3:e144. doi: 10.1002/mco2.144 35601657 PMC9115588

[86] Wang Y Chen R Wa Y Ding S Yang Y Liao J . Tumor immune microenvironment and immunotherapy in brain metastasis from non-small cell lung cancer. Front Immunol. (2022) 13:829451. doi: 10.3389/fimmu.2022.829451 35251014 PMC8891382

[87] Delbari P Ahmadvand MH Mirjani MS Hajikarimloo B Hamidi Rad R Kargar-Soleimanabad S . The safety and efficacy of anti-pd-1/pd-l1 monoclonal antibodies for lung cancer brain metastasis: A systematic review and meta-analysis on brain metastasis. Neurosurgical Rev. (2025) 48:253. doi: 10.1007/s10143-025-03418-z 39969599

[88] Negrao MV Skoulidis F Montesion M Schulze K Bara I Shen V . Oncogene-specific differences in tumor mutational burden, pd-l1 expression, and outcomes from immunotherapy in non-small cell lung cancer. J Immunother Cancer. (2021) 9. doi: 10.1136/jitc-2021-002891 34376553 PMC8356172

[89] Liu C Zheng S Jin R Wang X Wang F Zang R . The superior efficacy of anti-pd-1/pd-l1 immunotherapy in kras-mutant non-small cell lung cancer that correlates with an inflammatory phenotype and increased immunogenicity. Cancer Lett. (2020) 470:95–105. doi: 10.1016/j.canlet.2019.10.027 31644929

[90] Bar-Ephraim YE Kretzschmar K Clevers H . Organoids in immunological research. Nat Rev Immunol. (2020) 20:279–93. doi: 10.1038/s41577-019-0248-y 31853049

[91] Li H Harrison EB Li H Hirabayashi K Chen J Li QX . Targeting brain lesions of non-small cell lung cancer by enhancing ccl2-mediated car-t cell migration. Nat Commun. (2022) 13:2154. doi: 10.1038/s41467-022-29647-0 35443752 PMC9021299

[92] Scognamiglio G De Chiara A Parafioriti A Armiraglio E Fazioli F Gallo M . Patient-derived organoids as a potential model to predict response to pd-1/pd-l1 checkpoint inhibitors. Br J Cancer. (2019) 121:979–82. doi: 10.1038/s41416-019-0616-1 31666667 PMC6889147

[93] Zhou G Lieshout R van Tienderen GS de Ruiter V van Royen ME Boor PPC . Modelling immune cytotoxicity for cholangiocarcinoma with tumour-derived organoids and effector t cells. Br J Cancer. (2022) 127:649–60. doi: 10.1038/s41416-022-01839-x 35597867 PMC9381772

[94] Ballerini M Galiè S Tyagi P Catozzi C Raji H Nabinejad A . A gut-on-a-chip incorporating human faecal samples and peristalsis predicts responses to immune checkpoint inhibitors for melanoma. Nat BioMed Eng. (2025) 9:967–84. doi: 10.1038/s41551-024-01318-z 39939548 PMC12176660

[95] Baisiwala S Fazzari E Li MX Martija A Azizad DJ Sun L . A human tumor-immune organoid model of glioblastoma. Cell Rep. (2026) 45:116790. doi: 10.1016/j.celrep.2025.116790 41505256 PMC12969483

[96] Xu Y Chen K Xu Y Li H Huang Z Lu H . Brain radiotherapy combined with camrelizumab and platinum-doublet chemotherapy for previously untreated advanced non-small-cell lung cancer with brain metastases (c-brain): A multicentre, single-arm, phase 2 trial. Lancet Oncol. (2025) 26:74–84. doi: 10.1016/s1470-2045(24)00643-0 39756446

[97] Nounsi A Seitlinger J Ponté C Demiselle J Idoux-Gillet Y Pencreach E . Patient-derived tumoroid for the prediction of radiotherapy and chemotherapy responses in non-small-cell lung cancer. Biomedicines. (2023) 11. doi: 10.3390/biomedicines11071824 37509464 PMC10376341

[98] Bender T Schickel E Schielke C Debus J Grosshans DR Durante M . Aberrant choroid plexus formation drives the development of treatment-related brain toxicity. Commun Biol. (2025) 8:276. doi: 10.1038/s42003-025-07736-2 39987290 PMC11846864

[99] Pașca SP Arlotta P Bateup HS Camp JG Cappello S Gage FH . A framework for neural organoids, assembloids and transplantation studies. Nature. (2025) 639:315–20. doi: 10.1038/s41586-024-08487-6 39653126

[100] Spitz S Ko E Ertl P Kamm RD . How organ-on-a-chip technology can assist in studying the role of the glymphatic system in neurodegenerative diseases. Int J Mol Sci. (2023) 24. doi: 10.3390/ijms24032171 36768495 PMC9916687

[101] Du Y Li X Niu Q Mo X Qui M Ma T . Development of a miniaturized 3d organoid culture platform for ultra-high-throughput screening. J Mol Cell Biol. (2020) 12:630–43. doi: 10.1093/jmcb/mjaa036 32678871 PMC7751183

[102] Shrestha S Lekkala VKR Acharya P Siddhpura D Lee MY . Recent advances in microarray 3d bioprinting for high-throughput spheroid and tissue culture and analysis. Essays Biochem. (2021) 65:481–9. doi: 10.1042/ebc20200150 34296737 PMC9270997

[103] Ajongbolo AO Langhans SA . Brain organoids and assembloids-from disease modeling to drug discovery. Cells. (2025) 14. doi: 10.3390/cells14110842 40498018 PMC12155111

[104] Blee C Muthana M Wells G Danson S . Turning the tide: Harnessing vaccines and viruses to fight cancer. Immunotherapy Adv. (2025) 5:ltaf033. doi: 10.1093/immadv/ltaf033 41445691 PMC12723228

[105] Ning RX Liu CY Wang SQ Li WK Kong X He ZW . Application status and optimization suggestions of tumor organoids and car-t cell co-culture models. Cancer Cell Int. (2024) 24:98. doi: 10.1186/s12935-024-03272-x 38443969 PMC10916304

